# Crossing Paths in Human Renal Cell Carcinoma (hRCC)

**DOI:** 10.3390/ijms131012710

**Published:** 2012-10-05

**Authors:** Guadalupe Aparicio Gallego, Vanessa Medina Villaamil, Enrique Grande, Isabel Santamarina Caínzos, Luís M. Antón Aparicio

**Affiliations:** 1 Biomedical Research Institute (INIBIC), CHU A Coruña, Xubias de Abaixo s/n, PC 15006, A Coruña, Spain; E-Mails: lupe.aparicio@gmail.com (G.A.G.); Isabel.santamrina.cainzos@sergas.es (I.S.C.); 2 Medical Oncology Department, Ramón y Cajal Universitary Hospital, Madrid, PC 28050, Spain; E-Mail: egrande@oncologiahrc.com; 3 University of A Coruña (UDC), PC 15006, A Coruña, Spain; E-Mail: luis.m.anton.aparicio@sergas.es; 4 Medical Oncology Department, CHU A Coruña, PC 15006, Spain

**Keywords:** human renal cell carcinoma (hRCC), signaling pathway crosstalk, biomarkers

## Abstract

Historically, cell-signaling pathways have been studied as the compilation of isolated elements into a unique cascade that transmits extracellular stimuli to the tumor cell nucleus. Today, growing evidence supports the fact that intracellular drivers of tumor progression do not flow in a single linear pathway, but disseminate into multiple intracellular pathways. An improved understanding of the complexity of cancer depends on the elucidation of the underlying regulatory networks at the cellular and intercellular levels and in their temporal dimension. The high complexity of the intracellular cascades causes the complete inhibition of the growth of one tumor cell to be very unlikely, except in cases in which the so-called “oncogene addiction” is known to be a clear trigger for tumor catastrophe, such as in the case of gastrointestinal stromal tumors or chronic myeloid leukemia. In other words, the separation and isolation of the driver from the passengers is required to improve accuracy in cancer treatment. This review will summarize the signaling pathway crossroads that govern renal cell carcinoma proliferation and the emerging understanding of how these pathways facilitate tumor escape. We outline the available evidence supporting the putative links between different signaling pathways and how they may influence tumor proliferation, differentiation, apoptosis, angiogenesis, metabolism and invasiveness. The conclusion is that tumor cells may generate their own crossroads/crosstalk among signaling pathways, thereby reducing their dependence on stimulation of their physiologic pathways.

## 1. Introduction

Knowledge continues to increase regarding the intracellular cascades that govern the growth and spread of tumor cells. In recent decades, we have tried to inhibit tumor growth by selectively blocking the beginning of the cascade at the receptor level (such as in the use of anti-epidermal growth factor receptor (EGFR) inhibitors), blocking the middle of the cascade (such as with anti-RAF or anti-mTOR inhibitors) or blocking extracellular ligands (such as vascular endothelial growth factor (VEGF)) before contact with the transmembrane receptors expressed on tumor cells. Success in terms of a clear change in the natural history of the cancer is not frequently seen. We have only achieved long-term responses and stabilization of the disease in tumors in which a well-defined trigger starts the proliferation cascade, such as in the case of gastrointestinal stromal tumors (GIST) or chronic myeloid leukemia (CML). Even in these cases, curing the disease is not yet a realistic objective.

The barriers to cancer development are embodied in an ontogeny; cancer cells have defects in regulatory intracellular circuits that govern proliferation and homeostasis. The most complex mechanisms of acquired cancer cell autonomy derive from alterations in components of the downstream cytoplasmic circuitry that receives and processes the signals emitted by ligand-activated receptors.

These complex biological systems can be thought of as scale-free networks with an uneven distribution of connections to key signaling nodes. Signals downstream of pathways and other signals interact in a number of significant ways in the tumor tissues, acting in either a synergistic or antagonistic manner. Although some findings on pathway interaction may turn out to be cell type-specific, a general synthesis of the present state of knowledge can be attempted.

Unfortunately, signaling cascades are not linear and frequently interact, generating short-circuits between different intracellular pathways and increasing the complexity of the system. This complexity provokes many questions that are not yet understood. A fundamental paradigm in modern oncology is the discovery of biologically relevant pathways on which cancer cells rely for survival. This paradigm depends on multiple factors, including the identification of pathways that are truly critical for cell survival. Deciphering the added value of these and other signaling pathways will require a careful assessment of the risk and benefit of each pathway across different patient phenotypes.

Much remains to be learned about pathway crossroads as targets in human cancer with regard to inhibition with optimal risk/benefit ratios and the identification of novel targets for cancer therapy. The investigation of gene and protein expression will hopefully provide insight leading to the optimization of specific therapies for specific tumors. Translational investigation is needed to identify the molecular phenotypes associated with response and resistance to each of the agents.

This review provides a kaleidoscopic view of the last two decades of development in this field and the interactions between different cell pathways.

## 2. Signaling Pathways in hRCC

### 2.1. The Von Hippel-Lindau (VHL) Pathway

Von-Hippel-Lindau disease is a rare, autosomal dominant, familial cancer syndrome that manifests as retinal angiomas, hemangioblastomas of the central nervous system, pheochromocytomas and clear-cell renal carcinomas (ccRCC) [1]. VHL is caused by an inherited mutation of one allele of the *VHL* tumor suppressor gene located on chromosome 3q25-26 [2]. Associated focal lesions (e.g., renal cell carcinoma) are caused by inactivation or silencing of the remaining wild-type *VHL* allele. In sporadic clear cell renal carcinomas, *VHL* gene defects are common (60%–75%), and up to 20% of tumors exhibit decreased *VHL* expression due to hypermethylation [3–7]. Whether sporadic renal cell carcinoma (RCC) prognosis is related to the type of *VHL* mutation or altered expression is unclear. The *VHL* gene functions in the hypoxia-inducible pathway and the *VHL* gene product are a component of a multi-protein complex (consisting of Elongin B and C, Cul2, and Rbx1) that ubiquitinates the transcriptional factor hypoxia-inducible factor (HIF)-1α [8]. The Hypoxia-inducible factor (HIF)-1 complex, a heterodimer containing an α and β subunit, responds to hypoxic stress to regulate the expression of several genes, including *VEGF*, *EGFR*, platelet-derived growth factor (*PDGF*), glucose transporters (such as *GLUT-1*), transforming growth factor-α (*TGF-α*) and erythropoietin (*EPO*) [9,10]. *VHL* loss-of-function mutations prevent ubiquitin-mediated HIF-1α degradation, causing the upregulation of hypoxia-inducible genes. Growth factor and adhesion pathways (e.g., the RAS-mitogen activated protein kinase (MAPK) pathway and the phosphatidylinositol 3-kinase (PI3K)-AKT-mammalian target of rapamycin (mTOR) pathway) also regulate HIF-1α activity. In addition to HIF-1α, VHL binds to the cell-matrix protein fibronectin, the chaperonin TRiC/CCT, microtubules, and the transcription factor Jade-1, suggesting that these proteins also contribute to disease pathogenesis [1].

The two most important pathways in ccRCC are those governed by pVHL (Von Hippel-Lindau protein) and mammalian target of rapamycin complex 1 (mTORC1). It has been discovered that the REDD1 protein links these pathways [11].

### 2.2. The Notch Signaling Pathway

In recent years, *Notch* has emerged as a critical element in kidney development. Accumulating evidence suggests that Notch signaling plays a critical role in survival, proliferation, and cell fate at various stages of kidney development, including in the decision by kidney side population (SP) cells to self-renew or differentiate [12–14]. Although the existence of resident stem cells in the mature mammalian kidney has not been proven, SP cells in the adult kidney have been proposed to represent a progenitor population. The modulation of Notch signaling at various levels, including at the level of ligand expression, has provided evidence of the influence of this pathway.

A survey of the list of genes that are strongly expressed in kidney SP highlights many members of the Notch signaling pathway, including the receptors Notch1, Notch2, and Notch3, the ligands Jagged1 and Jagged2, the secreted protein radical fringe gene homologue (Rfng), the intracellular signaling molecules Deltex2 (Dxt2) and Deltex3 (Dxt3), the transcriptional regulators transducin-like enhancer of split (Tle1) and Riken (CBF1 interacting corepressor) and the noted Notch target genes *Gsk3b* [15] and *cyclins D1* and *E1* [16].

Notch1 and the Notch ligand Jagged1 are expressed at significantly higher levels in ccRCC tumors than in normal renal tissue [10]. The expression of Notch receptors has been shown to be deregulated in RCC. The expression levels of Notch1 and Notch 4 are significantly decreased in human renal cell carcinoma tissues as compared with adjacent non-neoplastic tissues. *Notch1* and *Notch4* are also markedly down-regulated in human renal cell cancer cell lines. In contrast, *Notch2* and *Notch3* expression is minimally detected [17].

### 2.3. The HIF Pathway

The hypoxia pathway (HIF) is crucial during developmental organogenesis and in several adult pathologies, such as cancer.

To maintain the appropriate level of ATP that is required for proper cellular metabolism, the oxygen concentration must be closely monitored [18]. During normoxia, the VHL tumor suppressor targets the hypoxia-inducible factor HIF-1α for ubiquitination and proteasomal degradation. In response to hypoxia, stabilized HIF-1α and HIF-2α proteins bind HIF-1β and initiate expression of genes that alleviate hypoxic stress, including those promoting neovascularization. Both HIF-1 and HIF-2 stimulate transcription of *VEGF*, a crucial regulator of vascular development. Tight regulation of the stability and subsequent transactivational function of HIF-1α is chiefly controlled by its post-translational modifications, such as hydroxylation, ubiquitination, acetylation, and phosphorylation.

For example, HIF-1α uniquely stimulates the expression of glycolytic enzymes, such as phosphoglycerate kinase and lactate dehydrogenase-A, carbonic anhydrase-9, and the pro-apoptotic gene *BNIP-3* [19–22]. In contrast, under hypoxia, HIF-2α up-regulates *CYCLIN D1*, *TFG-α*, and *EPO* [21,23–26]. A third group of genes, *VEGF*, and *GLUT1* are regulated by both α subunits [19,27].

These genes contain a hypoxia response element (HRE) in their regulatory sequences to which the HIF dimer binds. Overall, the activation of such hypoxia target genes enables cells to respond to oxygen deprivation by controlling angiogenesis, cell growth, and metabolism [28].

HIF-1α induces the expression of transforming growth factor α, with consequent activation of the epidermal growth factor receptor (EGFR)/phosphatidylinositol-3-OH kinase (PI3K)/protein kinase B (AKT)/IkB-kinase α (IKKα)/NF-kB signaling cascade [29].

HIF-2α stabilization can be observed by immunohistochemistry in renal interstitial cells [30]. HIF-2α is stabilized in von Hippel-Lindau (VHL)-deficient renal cell carcinoma through mechanisms that require ongoing mRNA translation. mTOR functions in two distinct complexes: Raptor-associated mTORC1 and Rictor-associated mTORC2. HIF-2α is frequently expressed in solid tumors. The role of HIF-2α in the pathogenesis of ccRCC has been the most extensively studied.

Activation of an autocrine signaling loop through TGF-α-mediated stimulation of the EGFR has been proposed to drive the serum-independent growth of renal carcinoma cells [25,31].

HIF-2α may also play a role in renal carcinomas caused by mutations in the tumor suppressor tuberous sclerosis-2 (*Tsc-2*) [32].

HIF-1α expression is low in 76% of RCC, and only 25% of patient samples display moderate to high staining. HIF-1α expression tends to be associated with better prognosis and might be less important than HIF-2α expression in human RCC [33].

Renal cell carcinoma, as is the case in many cancers, demonstrates oxidative stress. An interplay between hypoxia pathway and mTORC1 is that reactive oxygen species, such as hydrogen peroxide, activate AMP-activated protein kinase (AMPK)-alpha to inhibit mTORC1 signaling [34].

### 2.4. The VEGF Pathway

Six secreted glycoproteins comprise the *VEGF*-related gene family of angiogenic and lymphangiogenic growth factors, including *VEGF-A* (commonly referred to as *VEGF*), *VEGF-B*, *VEGF-C*, *VEGF-D*, *VEGF-E* and placenta growth factor (*PIGF*)-*1*. VEGF is an endothelial cell-specific mitogen *in vitro* and an angiogenic inducer of arteries, veins and lymphatic vessels *in vivo* [35–39]. As a pleiotropic growth factor, VEGF mediates multiple functions, including the regulation of vessel permeability, endothelial cell activation, survival, proliferation, invasion and migration. VEGF is implicated in developmental, reproductive, bone and pathological angiogenesis [37–40]. Located on chromosome 6p21.3, the human *VEGF-A* gene is organized into 8 exons and 7 introns [41]. The receptor tyrosine kinase FLT-1 (fms-like tyrosine kinase, VEGFR-1) and Flk-1/KDR (VEGFR-2) are high-affinity VEGF receptors. VEGF also interacts with neuropilins NP-1/2, a family of co-receptors [42,43]. VEGFR-3 is another member of the VEGFR-family that binds VEGF-C and D but not VEGF. VEGFR-2 mediates the majority of VEGF down-stream angiogenic effects, while VEGFR-1 is critical in developmental angiogenesis [37–39].

Tumor-associated stroma is another important site of VEGF production. VHL inactivation, which mediates the over-expression of VEGF in approximately 80% of ccRCC patients, causes VEGF and its receptor to be interesting targets for novel RCC treatment strategies [37–39].

A potential crosstalk of VEGF and MAPK in RCC angiogenesis is that VEGFR-3-mediated ERK signaling pathway contributes to lymphangiogenesis based on recent report [44].

### 2.5. The Epidermal Growth Factor Receptor (EGFR) Pathway

RCCs frequently show EGFR immunoreactivity [45,46] that is primarily localized to the cell membrane. In contrast, EGFR expression in normal renal tissues is chiefly observed in the cytoplasm. Previous studies have shown that up-regulation of *EGFR* is one of the common events in RCC tumorigenesis [47]. Over-expression of *EGFR* is thought to play an important role in the initiation and progression of RCC because up-regulation of *EGFR* has been associated with high tumor grade and worse prognosis [48,49].

Studies characterizing the localization of EGFR have shown that EGFR has membranous and cytoplasmic expression. EGFR membranous staining is significantly stronger in RCC tumors than in normal tissues. In contrast, EGFR cytoplasmic staining is significantly higher in normal than in tumor tissues [50]. The different locations of EGFR immunostaining may be associated with progression and prognosis in RCC [51,52]. A significant correlation also exists between the level of membranous EGFR expression and the histologic subtypes, with higher expression in conventional RCC than non-conventional RCC (including papillary, chromophobe, sarcomatoid, and collecting duct subtypes) [50]. Previous studies have indicated that cytoplasmic EGFR immunostaining is associated with higher tumor stage and grade and poor prognosis in RCCs [51,52]. However, the prognostic value of EGFR over-expression in RCC is a controversial issue. Some studies have shown an association between EGFR immunoreactivity and well-differentiated RCCs [52,53], whereas others have shown an association between EGFR immunoreactivity and high tumor stage/grade and poor prognosis [54] or no significant association at all [55].

Other studies have suggested the existence of a novel role for the EGFR signaling pathway, in which activated EGFR undergoes nuclear translocation and subsequently regulates gene expression to potentially mediate specific cellular processes [56,57]. This new role of EGFR is distinct from the well-known traditional EGFR signaling that involves the transduction of mitogenic signals through the activation of multiple signaling cascades.

Signaling through EGFR has been shown to result in NF-kB activation, and EGFR-mediated activation of NF-kB can occur in a PI3K/AKT-dependent manner in clear cell RRCs that constitutively express HIF-1α as a consequence of biallelic *VHL* loss [29].

Phosphorylation of the eukaryotic translation initiation factor 4E binding protein 1 (4EBP1) is abrogated by inhibition of the mTORC1, comprised of TOR, Raptor, and LST8, which is highly sensitive to inhibition by rapamycin [58]. Inhibition of mTORC1 is intricately regulated by the tuberous sclerosis Tsc-1/Tsc-2 protein complex [58]. Germline mutations in the Tsc-1 and Tsc-2 genes result in alterations in cell growth, survival, proliferation, migration, differentiation and angiogenesis [59]. The gene products of Tsc-1 (harmartin) and Tsc-2 (tuberin) form a heterodimeric protein complex that negatively regulates the mTOR signaling pathways [60]. Loss of tuberin expression results in increased levels of Rheb-GTP and subsequently activation of mTOR. Rheb-GTP/mTOR signaling has been implicated in tumor development, via its downstream modulation of protein synthesis, cell proliferation, cell cycle progression, and cell survival [58].

The Rafs regulate cellular proliferation and differentiation through the Raf/MEK/ERK/MAPK signaling cascade, which is hyperactivated in ~30% of all cancers [61]. In an *in vitro* kinase assay, recombinant 4EBP1 was a substrate for ERK/MAPK, suggesting that ERK may play a role in the hierarchical phosphorylation of 4EBP1. The loss of heterozygosis in the Tsc-2 gene and subsequent loss of tuberin expression observed in TGHQ-induced renal tumors [62], and tumorigenic QTRRE cells [63], makes this a unique model to study the role of constitutive Raf/ERK MAPK in tuberin-deficient renal carcinogenesis.

The study of the relationship between tuberin, the B-Raf/Raf-1/ERK MAPK cascade, and 4EBP1 hyperphosphorylation identified Raf-1 as an effective regulator of 4EBP1 phosphorylation and activator of cap-dependent translation during renal carcinogenesis. Inhibition of either the MAPK or mTOR pathway alone is insufficient to abrogate 4EBP1 phosphorylation because both mTOR and ERK are capable of modulating phosphorylation of 4EBP1 in renal proximal tubule cells [64].

### 2.6. The CAIX Pathway

Carbonic anhydrases (CA) are metalloenzymes that catalyze the reversible hydration of carbon dioxide to form bicarbonate and protons. The first reaction is catalyzed by carbonic anhydrase, and the second reaction occurs instantaneously.

In mammals, 16 different α-CA isoenzymes or CA-related proteins (CARP) have been described with very different catalytic activities and sub-cellular localizations. Some of these proteins are cytosolic (CA I, CA II, CA III, CA VII, and CA XIII), others are membrane-bound (CA IV, CA IX, CA XII, CA XIV, and CA XV), one is mitochondrial (CA Va and CA Vb) and one (CA VI) is secreted in saliva and milk. The different CAs have different tissue distributions, subcellular localizations, biological functions and kinetic properties [65].

Carbonic anhydrase IX (CA IX), or MN protein, consists of a signal peptide, a proteoglycan-related sequence, an enzymatically active extracellular carbonic anhydrase domain, a transmembrane segment and a short intracellular tail. CA IX is one of four transmembrane isoenzymes and has been implicated in the control of cell proliferation and cellular transformation [66].

CA IX overexpression has been identified in renal carcinoma, particularly clear cell adenocarcinoma [67]. CA IX has been well recognized to be a sensitive and specific marker of the clear cell histotype and is absent in papillary type, chromophobe and oncocytoma tumors [68–71]. RT-PCR analysis in benign and malignant renal tissue has demonstrated that the expression of *CA IX* is limited to ccRCC [72].

The expression of CA IX in advanced ccRCC has been described as an independent predictor of survival; decreased expression of CA IX portends a worse prognosis in patients with a metastatic clear cell histotype [73].

The fact that lower expression of CA IX is observed in metastatic lesions than in the corresponding primary tumor suggests that as tumors progress, they becomes less dependent on hypoxia-inducible factors and possibly become increasingly driven by mutations that control other pathways [74].

### 2.7. The GLUT 1-5 Transporters Pathway

Glucose metabolism is a central component of living systems. The oxidation of glucose represents a major source of metabolic energy for mammalian cells. However, tumor cells have a reduced capacity to use oxidative metabolism and rely instead on an increased rate of glycolysis and glucose utilization [75]. Increased active glycolytic metabolism is reflected in an increased rate of glucose uptake [76]. The increase in glucose uptake by malignant cells is currently accepted to be associated with the overexpression of glucose transporters (GLUTs). In malignant cells, this process is mediated by GLUTs, whose expression and activity is regulated by oncogenes and growth factors [77]. An increase in glucose transport and metabolism may reflect a requirement by these rapidly growing cells for additional sources of energy [78].

GLUT5 is over-expressed in most RCCs, but previous studies have shown that GLUT1 displays the highest expression in RCCs [79]. The elevated expression of GLUT5 could indicate a preferential utilization of fructose in RCC. This fructose utilization can be associated with the tumors’ need for an additional energy source.

Cancer cells maintain a high rate of glycolysis, even in the presence of oxygen (Warburg effect) [80], probably due to the utilization of fermentative metabolism under the hypoxic conditions that develop in more aggressive tumors. GLUT5 over-expression may allow an increase in fructose uptake and, conversely, the increase in fructose utilization in the tumor cells may lead to GLUT5 over-expression.

### 2.8. The p53 Pathway

p53 is a known inducer of apoptosis, and the prognostic significance of p53 in RCC remains controversial [81–84]. A wide variation in the incidence of *p53* mutations has been reported in RCC, and the prognostic significance of *p53* mutations for this tumor is unknown. Some authors have reported that *p53* mutations in RCC cases might be used as a prognostic factor, and they believe that over-expression of the mutant *p53* protein reflects the potential that genetic instability might have already occurred. In contrast to these reports, some studies have demonstrated that *p53* mutations have no value in predicting prognosis in RCC [81,82,85].

RCC rarely acquires mutations in the *p53* tumor suppressor gene (3% to 33%), suggesting that p53 signaling in this tumor type might be repressed by other mechanisms [86]. Loss of *p53* function is a critical event in the evolution of a tumor. This loss occurs through a range of molecular events, typically a missense *p53* mutation followed by loss of heterozygosity.

Although the best-studied mechanisms of p53 regulation are post-transcriptional [87], a less-appreciated (but nevertheless important) form of p53 regulation is at the level of *p53* transcription [88]. Recent studies have revealed that activation of Stat3 is associated with RCC progression and poor survival [89], while p53 induces apoptosis in renal tumor cells [90].

Also, signal transducer and activator of transcription Stat3 and p53 have been shown to integrate upstream signals and to be positive and negative regulators, respectively, of tumor cell proliferation. Stat3 and p53 also negatively regulate each other. These findings suggest that Stat3 and p53 are cooperatively involved in the development of RCC [91,92].

p53-independent apoptosis in RCC may due to regulation of MAPK pathway according to current research in a panel of tumor cells with mutant p53 [93].

### 2.9. The Transforming Growth Factor-β (TGF-β) Pathway

TGFs are peptides that reversibly promote anchorage-independent growth. The importance of TGF-β stems from the fact that it contributes to apoptosis control, angiogenesis, wound healing, immune regulation and tumor biology. TGF-β binds to the type II receptor, which then recruits and phosphorylates the type I receptor within its cytoplasmic domain [94]. The activated type I receptor then phosphorylates cytoplasmic substrates (the Smad proteins), which subsequently form complexes that translocate to the nucleus, thereby regulating the transcription of target genes [95].

Evidence for the critical role of TGF-β type I and type II receptors in TGF-β signaling and control of cell growth has been provided by studies of human neoplasia in which mutations in both type I and II TGF-β receptors are observed [96]. Reduced expression of the TGF-β type II receptor has been observed in RCC [97].

Signals from the activated TGF-β receptor complex are transduced to the nucleus by Smad proteins, a family of transcription factors [98]. To date, the Smads are the only TGF-β receptor substrates with a demonstrated ability to propagate signals. Reduced Smad2 and Smad4 expression has been observed in RCC. Reduced Smad2 expression in RCC correlates with a higher tumor grade, which is indicative of a more aggressive tumor [99].

TGF-β inhibits the proliferation of renal tubular epithelial cells and glomerular mesangial cells. TGF-β1 most likely inhibits cell growth by regulating the assembly and activity of cyclin-dependent kinase (cdk) complexes, which are necessary for cell-cycle progression from G_1_ to S phase [95,100,101].

In addition, TGF-β is clearly a master regulator of the immune response, and it exerts inhibitory effects on cells of all arms of the immune system, including Th1 cells, Th2 cells, CTLs, macrophages, NK cells, B cells and polymorphonuclear leukocytes (granulocytes). Importantly, TGF-β stimulates the production of connective tissue growth factor (CTGF), endothelin-1 and VEGF [102,103]; these factors collaborate in promoting the formation of a vascular and fibrous tumor stroma. Moreover, TGF-β attracts macrophages and other inflammatory cells to the stroma, and these cells secrete various mediators and growth factors that sustain tumor progression [104].

**Figure 1:**
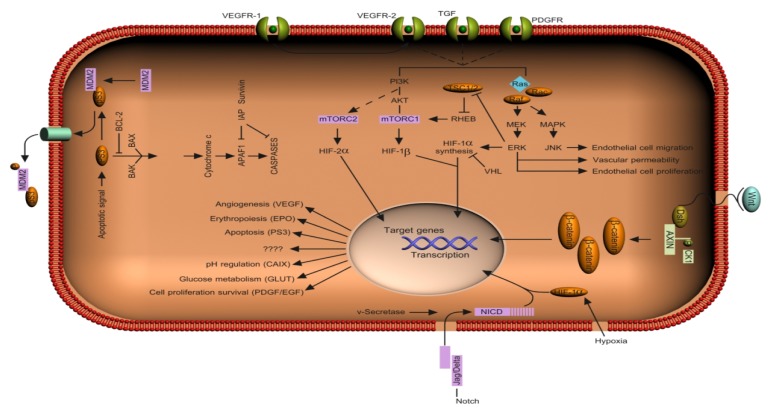
Schematic representation of selected signaling pathways in renal cell carcinoma (RCC). Angiogenic and cell proliferating signaling cascades are upregulated in RCC tumor cells. The vascular endothelial growth factor (VEGF) and other related growth factors secreted by tumor cells stimulate angiogenic signaling in the surrounding vascular endothelial cells. In response to growth factor signaling mediated through VEGF, platelet-derived growth factor (PDGF), and KIT receptors, PI3-kinase and Ras effectors activate hypoxia pathway (HIF) transcription factors, which in turn switch on gene expression needed for angiogenesis and cell proliferation in endothelial cells. In addition to the angiogenic pathway, the Wnt pathway is also upregulated in RCC tumor cells.

**Figure 2:**
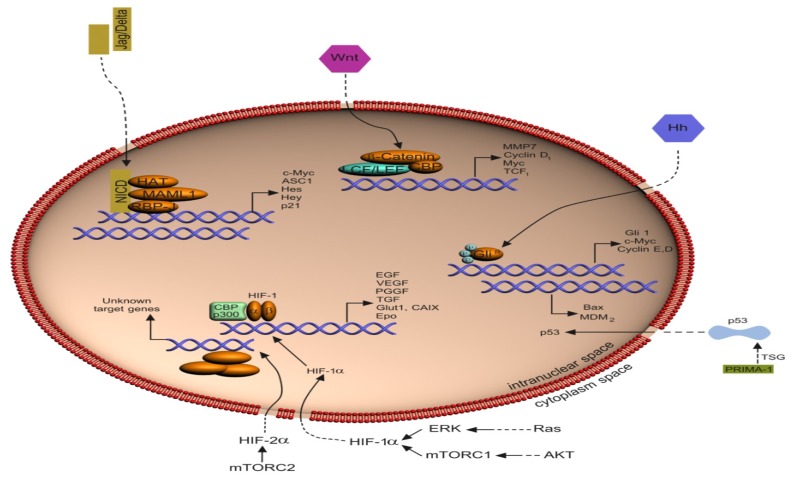
Nuclear targets of selected signaling pathways in RCC.

Upon tumor progression, TGF-β becomes a tumor promoter and induces an epithelial-tomesenchymal transition (EMT) through Smad-dependent and Smad-independent pathways. EMT is associated with increased secretion of matrix metalloproteinases, which promote tumor intravasation or extravasation. The effects of TGF-β on EMT, tumor growth or metastasis can be dissociated and might depend on different signaling pathways [105–107]. See schematics Figures 1 and 2.

### 2.10. The Transforming Growth Factor-α (TGF-α) Pathway

RCC produces cytokines, including transforming growth factor-alpha (TGF-α) [108,109], interleukin-6 (IL-6) [110], EGF [111], and insulin-like growth factor. Among these growth factors, TGF-α and IL-6 are produced at very high levels in RCC cells, suggesting that they play an important role in the proliferation of RCC.

## 3. Pathway Crosstalk

### 3.1. Crosstalk between the Notch Pathway and HIF Signaling

Hypoxia upregulates the expression of Notch targets. This upregulation might occur through the direct interaction with and the stabilization of Notch intracellular domain (NIC) by HIF-1α and/or by recruiting transcriptional coactivators [112]. Ultimately, the HIF-1α-NIC heterodimer binds to the promoters of Notch targets and synergistically increases transcription of genes involved in tumor progression and angiogenesis. Interestingly, hypoxia appears to enhance the stability of intracellular Notch.

Hypoxia induces the expression of the endothelial cell-specific Notch ligand Delta4, leading to increased Notch signaling [113,114].

Conversely, tumor hypoxia is a known link to increased metastatic potential, and Notch signaling is required to convert the hypoxic stimulus into an EMT, increased motility and invasiveness. Hypoxia-induced increased motility and invasiveness requires Notch signaling, and activated Notch mimics hypoxia in the induction of EMT. In this process, Notch signaling controls Snail-1 expression by two distinct but synergistic mechanisms [115].

### 3.2. Crosstalk between the Notch Pathway and EGFR Signaling

The tumor suppressor function of γ-secretase has been implicated in the mechanism that links the EGFR pathway and the Notch pathway. Notch is a well-recognized substrate of γ-secretase [116,117], and a reduction in γ-secretase activity results in altered Notch signaling by affecting the level of the transcription factor Hes1, a direct target gene that is up-regulated by Notch signaling.

Up-regulation of EGFR is dependent on the levels of γ-secretase activity, and a reduction in γ-secretase leads to up-regulation of EGFR. Therefore, the levels of EGFR appear to be inversely related to the levels of γ-secretase.

Consistent with these findings, whereas Notch signaling reduces EGFR activation, the up-regulation of EGFR is independent of Notch signaling, indicating that the EGFR pathway regulation by γ-secretase functions in parallel with Notch in tumorigenesis. Up-regulation of EGFR results from the down-regulation of Notch signaling.

Taken together, the activation of EGFR in parallel with altered Notch signaling plays a critical role in the tumor suppressor function of γ-secretase.

### 3.3. Crosstalk between the Notch Pathway and VEGF Signaling

Accumulating evidence has shown the intricate link between Notch activation and VEGF signaling. VEGF can induce the expression of Notch receptors and Dll4. Dll4 can reduce the expression of VEGFR-2 in endothelial cells (ECs), contributing to the feedback regulation of VEGF. Notch receptors have been shown to regulate the expression of endothelial VEGFRs [118].

Several studies have suggested that VEGF induces the expression of the Notch receptor via the PI3K-Akt pathway in human arterial endothelial cells to trigger Notch signaling, which plays a critical role in vascular formation during early embryonic development by activating the angiogenic process [119].

Notch induces VEGFR-3 expression *in vitro* in human endothelial cells and *in vivo* in mice [120]. The Notch/CBF1/suppressor of hairless/Lag-2 (CSL) complex binds and transactivates the VEGFR-3 promoter, providing evidence that the VEGFR-3 gene is a direct transcriptional target of Notch. Through induction of VEGFR-3, Notch signaling causes endothelial cells to become more responsive to VEGF-C (a VEGFR-3 ligand) and promotes endothelial cell survival and morphological changes. VEGF-C can bind and activate both VEGFR-2 and VEGFR-3, but Notch-mediated endothelial cell survival is most likely to occur via VEGFR-3 because VEGFR-2 expression is downregulated by Notch.

### 3.4. Crosstalk between the Notch Pathway and p53 Signaling

Crosstalk between the Notch pathway and the p53 pathway can occur at multiple levels in a manner that is dependent on the overall network organization of the individual cell types or tissues in which they operate [121–126]. Notch signaling can either suppress or increase p53 activity in a context-dependent manner that is closely connected to tumor promotion or suppression. Overall, the contexts in which Notch signaling restricts growth and/or induces apoptosis have been frequently linked with positive regulation of p53 activity and expression.

Notch activation has been shown to increase cell survival through the activation of the PI3K-Akt pathway, which leads to increased murine double minute 2 (MDM2) activity and consequent p53 degradation [127,128]. A second key function of Notch in transformation is the suppression of apoptosis through down-modulation of p53 [129].

In contrast, p53 can be positively affected in systems in which Notch activation exerts a growth-inhibitory or pro-apoptotic function (*i.e*., cervical carcinoma).

Notch and p53 are both expressed in RCC. Therefore, crosstalk between the pathways may exist at multiple levels, such that Notch and p53 can interact either positively or negatively depending on the cell type and stage of the cancer [130]. Notch signaling can regulate p53 activity, but p53 can also regulate Notch; these reciprocal positive or negative feedback loops are important for cell proliferation and cancer development [131].

Cross-regulation between the Notch and p53 pathways may also occur further down-stream, at the level of shared intermediates (*i.e.*, the transcriptional co-activator mastermind-like1 (MAML1)) [132,133] or an endocytic protein (NUMB) [134].

## 4. Conclusions

### 4.1. The Backbone of Intracellular Crosstalk

Each of the physiologic changes in the previously mentioned intracellular pathways represents the successful breaching of an anticancer defense mechanism that is hardwired into cells. This multiplicity may explain why cancer acquires autonomy and is largely due to the prevalence of dominant oncogenes that have been found to modulate tumor development. We suspect that the crosstalk between signaling pathways is deregulated in all human tumors. Although this point is difficult to prove rigorously the clues are abundant, and the appearance of secondary mutations, overexpression of other transmembrane receptors or activation of parallel intracellular pathways is frequently observed in oncology. We suggest that tumors may carry defects in many components of the signaling pathways. The nature of these alternative mechanisms for cell survival remains elusive. Under intensive study over years, the wiring diagram of the signaling circuitry crossroads in mammalian cells is coming into focus. New downstream signaling pathways that radiate from the cell membrane through the cytoplasm to the nucleus are being discovered with some regularity. These cascades are linked via a variety of cross-talking connections with other pathways, and these cross-connections enable extracellular signals to elicit multiple biological effects. The acquisition of crossroad signaling autonomy by cancer cells is conceptually satisfying. However, it is increasingly apparent that the deregulation of crossroads within a tumor cell can only be explained once we understand the contributions of the ancillary signaling pathways present in a tumor, which must play key roles in driving tumor cells. Heterotypic signaling inside the diverse cell types within a tumor may ultimately prove to be as important for explaining tumor cell proliferation. We suspect that many of the crossroad signals driving the proliferation of cancer cells originate from the cell components of the individual tumor cells.

### 4.2. The Hyper-Network Concept

Cells rely on a handful of core signaling pathways to guide a wide range of developmental processes, from the earliest specification events to more complex specialization functions. To achieve the morphological complexity that is characteristic of mammalian cells, these core signaling pathways must integrate to form a larger, complex signaling system, which we term the “hyper-network.” However, comprehensive knowledge of this network, the nodes that define it and its emergent properties are lacking. Studying how these highly pleiotropic pathways are interlinked is essential to understand development and evolution and, consequently, defines a fundamental problem in biology with obvious implications for cancer.

Because multiple mechanisms interlink diverse signaling pathways, the flexible nature of cross-talk may depend upon the primary mechanism activated in each context. However, a wide range of factors may also be involved, including additional crossroads. Through either a direct interface between pathways or shared target sets, signal integration might allow a simple interconnected system to generate an extraordinarily diverse output. The dramatic range of functions that these limited signals can create supports this paradigm. Studying its effects on the transcriptional output of integrated signals may be a useful approach to understanding the cross-talk.

Extrapolating from the signal cross-talk paradigm, several features of the signaling hyper-network can be inferred. First, signaling pathways are remarkably interlinked and can integrate at the cellular and multicellular level through varied mechanisms. Second, cross-talk is of broad importance, impacting numerous signaling pathways, and the regulation of the cross-talk appears to be deeply disturbed in cancer. Third, cross-talk is flexible, generating differing consequences in different type of tumors or in different contexts.

Thousands of positive and negative control feedback circuits inside the cells regulate the normal process of growth and proliferation. A disruption of this intracellular equilibrium is present in tumor cells and favors the activation of the anarchic proliferation and overproduction of growth factors that drive tumor development. Intracellular cross-talk occurs in every single solid tumor and is related to drug sensitivity and resistance, tumor escape and growth, prognosis, tumor behavior and multi-therapy resistance [135]. Several examples exist that demonstrate the role of intracellular cross-talk in solid tumors, such as those of the breast [136], lung [137], colorectal [138], and prostate [139].

In addition, cancer cells possess the adaptive ability to switch from one dominant growth factor pathway to another pathway under certain growth conditions to maintain proliferation and survival. In this sense, blocking one of the main signal transduction pathways may cause the tumor cells to switch their signal transduction to another non-blocked pathway to avoid the effects of the drug.

The link between different signaling pathways may be present—Not only at the intracellular level but also at the extracellular level—The location of the receptors. Indeed, one of the most studied intracellular cross-talk mechanisms that occur in cancer is the link between the epidermal growth factor receptor (erbB/HER) family and the insulin-like growth factor receptor (IGFR).

### 4.3. Targeted Therapies are the Option of Choice

The complexity of the aberrant signaling pathways in human cancer explains why interfering with only one single step of several that compose these pathways has not led to a sustained clinical response in cancer patients. Cancer cells have the ability to exploit diverse signaling pathways for growth advantage, cell survival and evasion of apoptosis and therefore by-pass the inhibition mediated by the administered drug. In fact, some of these alternative routes may even be facilitated by use of selective targeted agents and warrant interference at different stages to effectively reduce the tumor burden.

Furthermore, different etiological factors and risk habits can result in distinct genetic and epigenetic alterations, which may trigger the activation of different signaling pathways that impact development and progression of cancer.

The relatively limited effectiveness of traditional agents has led to the exploration of new, targeted drugs and to multi-targeted agents in cancer. Unfortunately, non-selective agents induce severe adverse events, and efficacy cannot currently be foreseen. The knowledge of cell biology and the mechanistic regulation of cancer are increasing and, together with the limited activity of conventional cytotoxic treatments, have allowed the development of new targeted therapies that thus far are not negligible and have shown a high percentage of disease stabilization and favorable safety profiles.

Signaling pathways that do not play a role in carcinogenesis may carry other molecular characteristics that can potentially be targeted.

Targeted therapy in oncology is based on the premise that tumor cells fundamentally rely on biological pathways to which drugs inhibiting those pathways can be applied. Biochemical studies of the signaling pathways deregulated in human cancer and target validation experiments have already culminated in the discovery and clinical application of small molecules with promising activity in cancer therapy.

Human cancer stands out among other pathologies with the identification of biologically relevant pathways and drugs that inhibit critical pathway elements with significant clinical effects. Cancer is highly heterogeneous with a complex combination of different types of cells with diverse features in the center or the margin of the tumor. Expression patterns are very sensitive and reflect the cellular composition of the whole tumor.

A fundamental paradigm of modern oncology is based on the discovery of the biologically relevant pathways that the cancer cell is dependent upon for survival. Drugs that inhibit one or more elements of the pathway are administered, ultimately leading to cancer cell death. This paradigm is dependent on multiple factors, including the identification of pathways that are truly critical for cell survival. Furthermore, drugs must effectively inhibit these pathway elements, and this inhibition must result in cell death.

Cancer clearly comprises a diverse biologic array of diseases with different pathways that are relevant to tumor differentiation and growth. The investigation of gene and protein expression will hopefully provide insight that will lead to the optimization of specific therapies for specific tumors. Clinical testing of combination or sequential administration of targeted agents has begun, encompassing all possible combinations of active agents. This concept is predicated on the premise of modern oncology that combinations of non-cross-resistance agents can be combined for therapeutic benefit, such as an increased cure rate. The initial clinical results suggest intolerance to some combinations, tolerance at less than full doses to others, and few able to be tolerated at full doses of both agents. Some promising clinical efficacy has been observed and is balanced against increased toxicity. For such therapy to advance in the treatment of human cancer, a combination must have greater clinical benefit than sequential monotherapy of the same agents. At present, combinations of targeted therapies remain investigational.

To date, only the HIF/VEGF and mTOR pathways have been exploited for therapeutic purposes in RCC and, despite the sound preclinical rationale, targeting the EGFR pathway resulted in almost no clinical results. Despite the improvement made with agents targeting key molecular pathways, cure of RCC is still out of sight; furthermore, to date, those few cases of metastatic RCC patients who have been cured of their advanced disease have received immunotherapy.

Much remains to be learned about signaling pathways as targets in human cancer, including inhibition with optimal risk/benefit ratios and the identification of novel pathways and targets. Translational investigation is needed to identify the molecular phenotype of response and resistance to each of the therapeutic agents.
